# Telecom-band multiwavelength vertical emitting quantum well nanowire laser arrays

**DOI:** 10.1038/s41377-024-01570-7

**Published:** 2024-09-04

**Authors:** Xutao Zhang, Fanlu Zhang, Ruixuan Yi, Naiyin Wang, Zhicheng Su, Mingwen Zhang, Bijun Zhao, Ziyuan Li, Jiangtao Qu, Julie M. Cairney, Yuerui Lu, Jianlin Zhao, Xuetao Gan, Hark Hoe Tan, Chennupati Jagadish, Lan Fu

**Affiliations:** 1 Frontiers Science Center for Flexible Electronics, Xi’an Institute of Flexible Electronics (IFE) and Xi’an Institute of Biomedical Materials & Engineering, Northwestern Polytechnical University, 127 West Youyi Road, 710072 Xi’an, China; 2 Department of Electronic Materials Engineering, Research School of Physics, The Australian National University, Canberra, ACT 2600 Australia; 3 Key Laboratory of Light Field Manipulation and Information Acquisition, Ministry of Industry and Information Technology, and Shaanxi Key Laboratory of Optical Information Technology, School of Physical Science and Technology, Northwestern Polytechnical University, 710129 Xi’an, China; 4 Australian Centre for Microscopy and Microanalysis, the University of Sydney, Sydney, NSW 2006 Australia; 5 School of Engineering, College of Engineering, The Australian National University, Canberra, ACT 2600 Australia; 6 ARC Centre of Excellence for Transformative Meta-Optical Systems, Research School of Physics, The Australian National University, Canberra, ACT 2600 Australia

**Keywords:** Nanowires, Semiconductor lasers

## Abstract

Highly integrated optoelectronic and photonic systems underpin the development of next-generation advanced optical and quantum communication technologies, which require compact, multiwavelength laser sources at the telecom band. Here, we report on-substrate vertical emitting lasing from ordered InGaAs/InP multi-quantum well core–shell nanowire array epitaxially grown on InP substrate by selective area epitaxy. To reduce optical loss and tailor the cavity mode, a new nanowire facet engineering approach has been developed to achieve controlled quantum well nanowire dimensions with uniform morphology and high crystal quality. Owing to the strong quantum confinement effect of InGaAs quantum wells and the successful formation of a vertical Fabry–Pérot cavity between the top nanowire facet and bottom nanowire/SiO_2_ mask interface, stimulated emissions of the EH_11a/b_ mode from single vertical nanowires from an on-substrate nanowire array have been demonstrated with a lasing threshold of ~28.2 μJ cm^−2^ per pulse and a high characteristic temperature of ~128 K. By fine-tuning the In composition of the quantum wells, room temperature, single-mode lasing is achieved in the vertical direction across a broad near-infrared spectral range, spanning from 940 nm to the telecommunication O and C bands. Our research indicates that through a carefully designed facet engineering strategy, highly ordered, uniform nanowire arrays with precise dimension control can be achieved to simultaneously deliver thousands of nanolasers with multiple wavelengths on the same substrate, paving a promising and scalable pathway towards future advanced optoelectronic and photonic systems.

## Abstract (web summary)

An innovative multi-step facet engineering approach was developed to achieve InGaAs/InP quantum well nanowire arrays with conttolled dimensions, uniform morphology and high crystal quality, enabling low-threshold lasing in the telecom-band.

## Graphical abstract

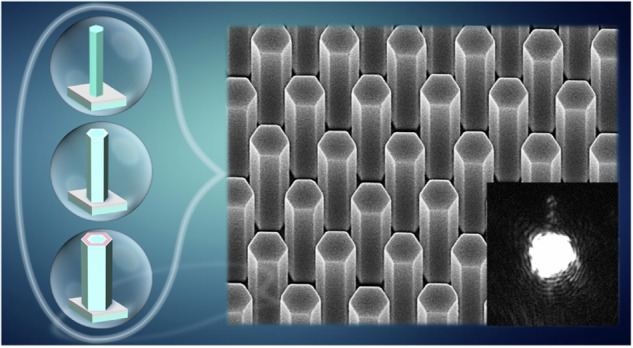

## Introduction

Semiconductor nanowires (NWs) offer compact, cost-effective, and low-threshold nanoscale lasers, ideal for applications in optical interconnects, medical diagnosis, and super-resolution imaging^1–11^. In particular, telecom-band NW lasers hold promise for on-chip coherent light sources in photonic integrated circuits, driving innovations in optical and quantum communication and computing^12–24^. To realize high-performance telecom-band NW lasers, it is imperative to focus on efficient gain media, optimal gain range, and effective optical cavity design^25^. This necessitates the epitaxial growth of high-quality NWs with smooth sidewalls, controlled dimensions, and precise crystal composition. Core–shell NWs with radial multiple-quantum wells (MQW) are attractive candidates due to their large active regions, tunable bandgap energy and quantum confinement effect, which are highly desirable for in nanoscale lasers.

However, epitaxial growth of MQW structures with both good structural and optical properties, along with uniform morphology, has proven to be a significant challenge. For instance, MQW NWs grown using the vapor-liquid-solid (VLS) method, such as GaAs/AlGaAs^26^ and InGaAs/InP^27^, exhibit tapering and nonuniform morphologies. This leads to suboptimal optical confinement and a low-quality (Q) factor for the NW cavity. Selective area epitaxy (SAE) offers the potential to control QW NW morphology on various substrates^28–31^. However, achieving high crystallinity in GaAs-based NWs through the SAE technique remains problematic, resulting in defects like twinning and planar defects^32^. This leads to non-radiative recombination centers and degraded optical properties. Additionally, pure GaAs NWs suffer from high density of surface states, making room temperature lasing difficult without additional passivation steps^8,12,33^. In contrast, high-quality InP-based NWs with ultralow surface non-radiative recombination rates can be conveniently achieved using SAE technology, allowing for room temperature lasing^29,34–36^. But, many SAE-grown InGaAs/InP MQW NWs are based on wurtzite (WZ) crystal structure^37,38^, which exhibit complicated facet evolution and severe morphology deterioration, rendering them unsuitable for lasing due to their asymmetrical shape. By adjusting the growth window to low-temperature and high V/III ratio, highly uniform InGaAs/InP MQW NWs can be grown from InP NWs in a mixed zincblende (ZB) and WZ phase, which leads to the demonstration of room temperature optically pumped lasing at a wavelength of ~1 µm^39^. However, polytypic NWs also suffer from a high density of stacking faults and twin-plane defects, resulting in substantial internal optical loss. Moreover, the maximum achievable length of these polytypic InGaAs/InP MQW NWs is less than 2 µm due to limited axial growth under low-temperature and high V/III ratio conditions^39^. Such short NW lengths are more sensitive to temperature variations, which can lead to fluctuations in the lasing wavelength and reduced spectral purity of the laser output. In addition, the utmost importance lies in the direct growth of high-density NW lasers characterized by meticulously controlled sites and a flawlessly pristine surface, free from any processing damage. This precision in site control, coupled with an unblemished surface, enhances the practical significance of these lasers, rendering them indispensable for the seamless integration of photonic chips on a larger scale. While Chang et al. have explored diverse emission directions to achieve this objective^40,41^, the existing challenge lies in the lack of precise nanowire morphology control, preventing the attainment of high-quality optical cavities necessary for optical mode control. Thus, an effective growth strategy remains elusive for obtaining InGaAs/InP MQW NWs with high uniformity, crystal quality, and dimension controllability, which is crucial for efficient NW lasing.

In this work, we introduce an innovative multi-step facet engineering approach for WZ based InGaAs/InP MQW NW growth via SAE method. A $\left\{1\bar{1}00\right\}$ faceted WZ InP NW core was firstly grown to the desired length under high-temperature and low V/III ratio conditions^42^ followed by changing the growth conditions to low temperature and high V/III ratio to promote lateral InP shell growth with a 30° rotation of all NW sidewalls, transitioning from $\left\{1\bar{1}00\right\}$ to {$11\bar{2}0$} orientation^43–45^. This allows for the subsequent InGaAs/InP MQW growth with a well-maintained hexagonal shape and smooth NW morphology, which is critical to facilitate the formation of a high-*Q* factor vertical Fabry–Pérot (F–P) cavity for MQW lasing in the vertical direction. Single mode vertical emitting laser centered at 1532 nm has been achieved at room temperature with a low threshold power of ∼28.2 μJ cm^−2^ per pulse and a high characteristic temperature of 128 K. By tuning the indium composition of the MQWs, tunable lasing peak has been achieved from 940 nm to telecommunication O and C band. Finally, simultaneous lasing is demonstrated from a substantial number of NWs in the same array, offering a promising path toward large-scale nano-laser integration.

## Results

**Fig. 1:**
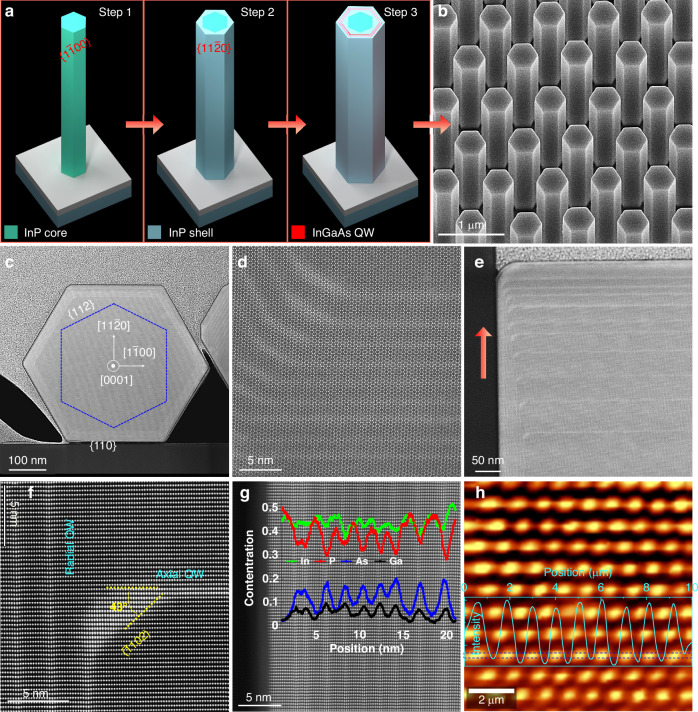
Structural and optical characterization of the MQW NW array. **a** Schematics of the WZ based InP NW, facet-rotated InP core–shell NW, and InGaAs/InP MQW NW, respectively. **b** 30° tilted SEM image of the NW array grown on InP(111)A substrate. **c**, **d** STEM-HAADF image of the lateral cross-section of an InGaAs/InP QW NW at different magnifications. The dashed blue line in (c) indicates the expected position of WZ InP NW core which is covered with the 30° rotated InP shell and MQW structure. **e**–**g** STEM-HAADF image taken along [112] zone axis from the vertical cross-section of NW top segment. The pink arrow in (**e**) indicates the NW growth along the (111)A direction. Line scan of EDX intensity along the radial QWs are superimposed in (**g**). **h** PL intensity map of the NW array with the intensity line scan from the region within the blue dashed lines showing uniform optical emission from the array

To ensure sufficient optical gain and efficient radiative recombination with good optical confinement, a multi-step growth by selective area metal organic vapor phase epitaxy was developed. The NW array size is 200 ×200 μm with a pitch size of 800 nm and opening hole diameter of 120 nm. Firstly, core InP NWs are grown at a high temperature and low V/III ratio condition to obtain high crystal quality WZ NWs^42^, with a length of 4 μm. Then the growth conditions are switched to low temperature and high V/III ratio condition to enable uniform radial InGaAs/InP MQW growth^43–45^, which is schematically shown in Fig. 1a. Figure S1a, b present the schematic lateral/vertical cross-section and SEM images of WZ InP NW core, which is grown at 680 °C with a V/III ratio of (297), exhibiting a taper-free hexagonal cross-section with six $\left\{1\bar{1}00\right\}$ sidewalls, as previously reported pure WZ-phase InP nanostructures^29,30^. Based on the $\left\{1\bar{1}00\right\}$ faceted WZ InP NW core, an InP shell was grown at 610 °C with V/III ratio ~ 4200, with the schematic cross-section and SEM image of the core–shell structure shown in Fig. S1c, d, presenting a similar hexagonal shape with a {1$1\bar{2}$0} NW facet 30° rotated from the original $\left\{1\bar{1}00\right\}$ NW facet and an enlarged diameter due to the enhanced lateral growth. Compared to WZ core-only NWs shown in Fig. S2, the lifetime of InP core–shell NWs is greatly extended, indicating a significant improvement in optical properties, which could be attributed to the reduced surface recombination velocity in this core–shell-like structure^46^. More detailed comparison of WZ InP NW, mixed ZB/WZ InP NW and WZ InP core–shell NW can be found in Table S1.

The excellent structural and optical properties of the WZ core–shell InP NWs form a good base for the subsequent InGaAs MQW incorporation (see Fig. S2). Following the growth of InP core–shell NWs, a 10-QW InGaAs/InP structure was sequentially grown with the detailed growth condition provided in Table S2. The highly uniform morphology of the 10-QW NWs shown in Fig. S1e, f indicates a conformal MQW structure growth without any facet transition or morphology deterioration. The MQWs have the same sidewall orientation as the InP core–shell NW, confirming that {1$1\bar{2}$0} faceted NW can work as an ideal platform for MQW growth, in sharp contrast to $\left\{1\bar{1}00\right\}$ faceted NW based MQW structure with asymmetrical morphology and complex facet evolution^38,47^. Compared with ZB/WZ mixed phase {110} faceted InGaAs/InP MQW NWs^37^, the WZ core–shell InP based MQW NWs offer more freedom in dimension engineering desirable for high-quality optical cavity design to modulate lasing peak and optical mode, as their length can be controlled by tuning the growth time of WZ InP NW core and diameter can be finely adjusted by changing the growth time of InP NW shell and MQW structures. Furthermore, defect-free or very few stacking faults can be achieved in WZ InP NWs, in comparison to the defective polytypic InP NW core, to enable higher crystal quality MQW growth for lasing. A detailed summary of various InGaAs/InP QW NWs achieved so far under different growth strategies is presented in Table S3, showing the great advantages offered by this new facet engineering strategy for high-quality InGaAs/InP MQW NW growth.

The micro-structural analysis of the InGaAs/InP 10-QW NW was performed by scanning transmission electron microscopy (STEM) on lamellas prepared by focused ion beam (FIB) cross-sectioning. Figure 1c, d presents the high-angle annular dark-field (HAADF) images of the lateral cross-section of the NW at different magnifications. The InGaAs MQW layers can be distinguished as brighter rings due to the atomic mass difference induced Z-contrast, clearly showing the coaxial and symmetrical arrangement of alternating hexagonally shaped QWs and barriers. For this particular NW, the NW diameter is around 500 nm, and QW thickness is less than 2 nm. It is worth mentioning that the diameter of the NWs can be adjusted by pitch size, opening hole diameter, and the growth time. The QWs at the six corners of NW are $\left\{1\bar{1}00\right\}$ faceted, and are relatively thicker compared to the QWs on {1$1\bar{2}$0} sidewalls, which is likely due to the larger atomic diffusion at the corners of the NWs^48^. While the $\left\{1\bar{1}00\right\}$ faceted sidewalls have a very short length (< 5 nm), and gradually diminish with increased growth time, they do not affect overall NW morphology. Figure 1e and Fig. S3 show the vertical cross-sectional image of NW top segment along the NW growth direction. Both radial and axial QWs can be identified as brighter lines, with the alternating QWs and barriers continuously covering the {1$1\bar{2}$0} sidewalls. On the other hand, the thickness of the axial and radial QWs significantly reduces with the growth time, as a result of the gradual increase of NW diameter as well as a reduction in the axial growth rate at low temperature. High resolution HAADF images in Fig. 1f and Fig. S3b reveal a small ($\bar{1}102$) facet at the top corner of the WZ InP NW core, where the axial QWs merge with radial QWs and subsequently fill the conjunction region. The QW chemical composition analysis was performed by energy dispersive X-ray spectroscopy (EDX). Figure S5–S7 shows the EDX maps of In, As, Ga and P elements at the top region from different NWs, clearly showing the presence of Ga and As in the QWs. For quantitative analysis of the chemical composition, EDX line-scans were performed across both the axial and radial QWs, and the results are superimposed with the HAADF image in Fig. 1g. Compared with the InP barrier layers, all the QW layers show a decreased In concentration commensurate with the increased Ga concentration and contain a P concentration due to interdiffusion effect. The average chemical composition for radial QWs is estimated to be In_0.85_Ga_0.15_As_0.4_P_0.6_.

Via this growth strategy, InGaAs/InP MQW NWs can be grown with highly uniform morphology, arranged in ordered arrays, with controlled length and diameter, as shown in Fig. 1b and Fig. S1f. The MQWs in different NWs within the same array appear to be relatively uniform, as seen in Fig. S4. The optical properties of a single MQW NW were characterized by cathodoluminescence (CL) and PL spectroscopy at room temperature. Figure S8a presents the SEM and panchromatic CL image acquired by an InGaAs detector (wavelength coverage: 1–1.6 µm), showing strong emission from the MQW region due to the high quality of InGaAs QWs and InP barriers along the whole NW. Figure S8b presents the representative PL spectra measured on the top of NW array, showing the strong broadband emission covering 1.1–1.9 µm wavelength range, due to the contribution from both the axial and radial QWs, as well as the geometry/composition variance between the axial and radial InGaAs MQWs. The lifetime of QW emission is extracted to be ~0.25 ns, which is much lower compared with the lifetime of InP core–shell NW, indicating enhanced carrier recombination due to the quantum confinement effect (Fig. S8c). In addition, the uniform and bright luminescence from ~100 NWs of the array is further verified by the PL intensity mapping as well as the line scan shown in Fig. 1h, indicating their great promise for large-scale device applications. It should be noted that, owing to the large lateral growth of the InGaAs MQWs and InP barriers, the NW diameter is much larger (>400 nm) than that of the SiO_2_ opening (~120 nm), creating an effective refractive index contrast at the NW/SiO_2_ interface for the formation of a vertical F-P cavity for each NW in the array which is attached to the substrate.

**Fig. 2:**
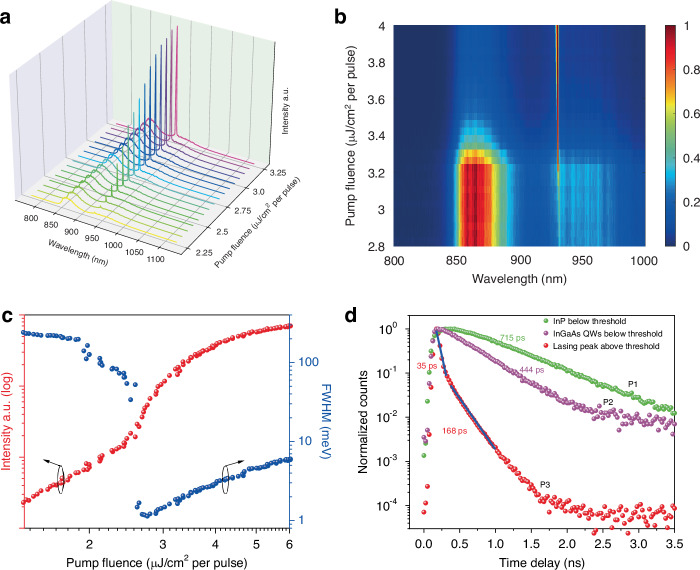
Lasing characterization of a single on-substrate NW. **a** Emission spectrum at different pump fluences. **b** Normalized emission intensity spectral map as a function of pump fluence. **c** Lasing emission intensity (red) and the corresponding FWHM of the spectrum (blue) as a function of pump fluence plotted on a logarithmic-logarithmic scale. **d** Normalized time-resolved emission decays from the InP, InGaAs QWs and lasing peak at different pump fluences

To evaluate the lasing properties, optical pumping of individual on-substrate NWs with a diameter of ~405 nm from another array was conducted in a home-made confocal micro-PL system at 5 K. Figure 2a shows the emission spectra from a single NW under different pump fluences. At low pump fluences, two peaks appear in the broad PL spectrum, which can be ascribed to emission from InP (865 nm) and InGaAs QWs (950 nm). When the pump fluence is increased to 2.5 μJ cm^−2^ per pulse, the QW peak becomes narrower and shifts to a shorter wavelength at 930 nm. As the pump fluence continues to increase, this narrow peak intensifies rapidly and dominates the entire emission spectrum while the spontaneous emission is clamped (see Fig. 2b). The transition process from the spontaneous emission to the amplified spontaneous emission (ASE), and to the stimulated emission with increased pump fluence is verified by the typical “*S*”-shape of the L–L curve (light output *versus* light input curve) shown in Fig. 2c. The corresponding full width at half maximum (FWHM) as a function of pump fluence is also displayed as blue dots in Fig. 2c. It can be found that the gain is not sufficient to dominate over the entire spectrum, and spontaneous emission dominates at low pump fluences, resulting in a broad emission spectrum; As the pump fluence increases, more carriers are excited to higher energy states, achieving population inversion. As the pump power further increases, the population inversion grows and the gain of the medium increases. The gain of the lasing mode is typically highest at the peak of the gain spectrum. As the gain increases, the feedback mechanism of the laser preferentially amplifies the wavelengths near the peak of the gain spectrum more than those at the wings, resulting in spectral narrowing. Then, a dramatic drop in FWHM can be clearly seen at threshold, indicating that single mode lasing with a low lasing threshold *P*_th_ of 2.7 μJ cm^−2^ per pulse is achieved in this vertically standing NW.

To evaluate the luminescent efficiency of these MQW NWs, time-resolved PL decay measurements are carried out for the various peak positions of the spectra under different excitation fluences, as shown in Fig. 2d. Figure S9 shows the different peaks originate from the InP centered at 865 nm (P1), spontaneous emission from InGaAs QWs centered at 955 nm below lasing threshold (P2), and lasing peak centered at 930 nm above threshold (P3), respectively. Figure 2d shows two mono-exponential decays and one double-exponential decays corresponding to P1, P2 and P3, respectively. Accordingly, the spontaneous emission lifetimes can be estimated as 715 ps for the InP and 444 ps for InGaAs QWs by fitting the time decay plots of P1 and P2 with a mono-exponential curve^49^. The difference in the carrier lifetime indicates that the radiative recombination rate of carriers in the QWs is faster than that in the InP due to quantum confinement effect^49,50^. Above the lasing threshold, the time decay of the P3 comprise a resolution-limited stimulated emission lifetime ~ 35 ps in the early stages and a longer spontaneous emission lifetime ~168 ps of the at the later stage, indicating that the former dominates the entire PL spectrum. All the results show that these core–shell MQW NWs have achieved excellent carrier confinement effect, enabling high radiative recombination efficiency and lasing.

**Fig. 3:**
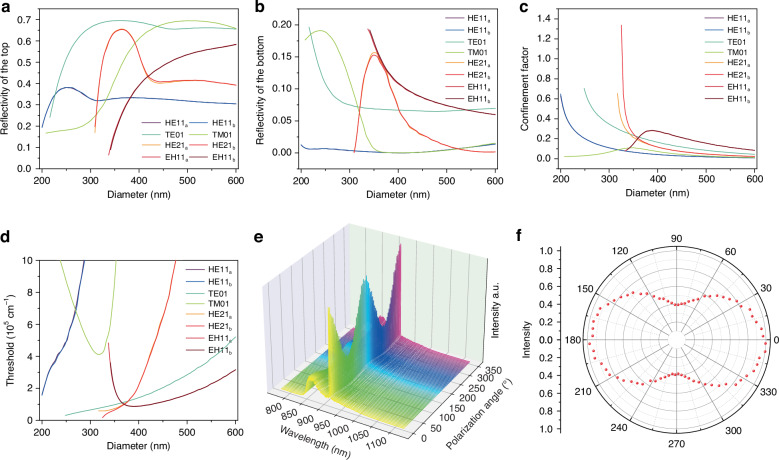
Mode identification through 3D FDTD simulation. Top (**a**) and bottom (**b**) surface reflectivity, confinement factor (**c**) and the calculated threshold gain (**d**) versus NW diameter for each transverse mode. **e**, **f** Polarization dependence emission spectra (**e**) and the lasing intensity polar plot (**f**) measured from a MQW NW

To understand the lasing mode of these InGaAs/InP MQW NWs, a threshold gain analysis for different NW diameters was carried out using finite difference time domain (FDTD) simulation. Around the lasing peak (930 nm), the threshold gains of the possible lasing modes (HE_11a_, HE_11b_, TE_01_, TM_01_, HE_21a_, HE_21b_, EH_11a_, EH_11b_) are calculated according to^14,33,51,52^

$${g}_{{\rm{th}}}=\frac{1}{\varGamma L}{\mathrm{ln}}\left(\frac{1}{\sqrt{{R}_{1}{R}_{2}}}\right)$$

where *Γ* is the confinement factor, *L* is the length of the NW, *R*_1_ and *R*_2_ are the reflectivity of the top and bottom facets of the standing NW, respectively. In this vertically standing F-P cavity, the top mirror is formed by the InP/air interface and the bottom mirror is formed by the InP/SiO_2_ interface. The reflectivity of the top facet of the vertical NW is plotted in Fig. 3a for different modes. For the bottom mirror, the NW core with a diameter of 120 nm is in direct contact with the substrate. Because the majority of the energy of the HE_11a_, HE_11b_, and TM_01_ modes is concentrated at the central axis of the NW, they leak into InP substrate. Therefore, the reflectivity of these modes is reduced significantly. The TE_01_, HE_21a_, HE_21b_, and EH_11a_ and EH_11b_ modes on the other hand, have the majority of their energy distributed at the periphery of the NW, resulting in relatively high reflectivity because of the interface with SiO_2_, as shown in Fig. 3b. The bottom surface reflectivity can be further increased by depositing a thicker SiO_2_ layer to lower the lasing threshold (see Fig. S10). After calculating the optical confinement factors *Γ* (see Fig. 3c), the curves of threshold gain versus NW diameter can be obtained, as plotted in Fig. 3d. For the NW with a diameter of 405 nm as studied experimentally in this work, the lowest threshold modes are the two degenerate transverse EH_11a_ and EH_11b_ modes. To confirm the simulation result, polarization dependence of the emission spectrum is further conducted. From the lasing intensity versus polarization angle plot shown in Fig. 3e, f, it can be verified that this lasing mode is linearly polarized with an extinction ratio $\rho =\left({I}_{\parallel }-{I}_{\perp }\right)/\left({I}_{\parallel }+{I}_{\perp }\right)$ of 45%. By comparing the electric field distributions of the possible supported modes shown in Fig. S11, the polarization dependence is in good agreement with the EH_11a/b_ mode.

**Fig. 4:**
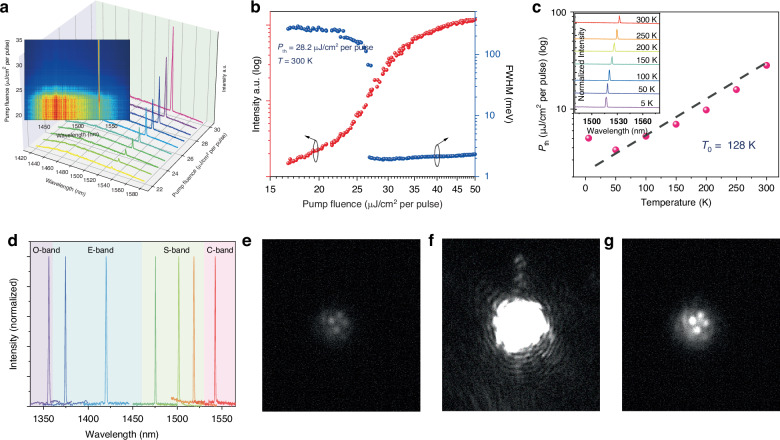
Telecom-band lasing characterization. **a** Emission spectra at different pump fluences of a single standing NW. Inset shows the corresponding normalized spectral intensity map. **b** Emission intensity (red) and the corresponding FWHM (blue) of the spectra as a function of pump fluence. **c** Lasing threshold versus operation temperature. The dashed line is a fitting of the experimental data to extract the characteristic temperature. Inset shows the lasing spectrum at various temperatures. **d** Telecom-band lasing spectra from NWs with different indium compositions in the QWs at room temperature under a pump fluence of 1.3 *P*_th_. **e**, **f** Optical images from the NW array before (**e**), and after (**f**) lasing threshold. **g** Image on (**f**) under an attenuated pump fluence

High-performance telecom-band nanoscale lasers are desirable for silicon-based on-chip optoelectronic integrated circuits due to the low transmission loss. To further extend the lasing wavelength to meet this important application requirement, InGaAs/InP MQW NW arrays were grown with different indium compositions in the QWs. Figure 4a shows the emission spectra from a single NW of the array samples. With an increasing pump fluence, the InGaAs QW peak can be seen evolving from a broad PL emission to single mode lasing centered at 1532 nm at room temperature with a low threshold power of ∼28.2 μJ cm^−2^ per pulse, as observed in the spectral intensity map in the inset of Fig. 4a. The typical “*S*”-shape in L-L curve and the variation of FWHM with increasing pump power illustrates the stimulated emission process of the NWs, as shown in Fig. 4b. The plot of lasing threshold as a function of operating temperature is shown in Fig. 4c, where the lasing peak is slightly redshifted from 1516.9 to 1532.4 nm with temperature due to the changes of the bandgap and refractive index (see the inset of Fig. 4c and Fig. S12). Figure S12 shows the lasing spectra and the corresponding L-L curves under different operating temperatures. By fitting the data in Fig. 4c with ${p}_{{\rm{th}}}={e}^{t/{t}_{0}}$, the characteristic temperature is estimated to be 128 K, which is comparable with those of the reported for horizontal NW lasers^33,49,51^, indicating good temperature characteristics despite without any heatsinking. Lasing peak tunability in the telecom-band is another important requirement for optical communication systems. By changing In composition in the InGaAs QWs lasing in the telecommunication bands, including O-band, E-band, S-band and C-band, can be achieved as shown in Fig. 4d from 1356 to 1542 nm. The corresponding power-dependent lasing spectra can be found in Fig. S14. To the best of our knowledge, this is the first report on lasing from a single NW vertically standing in a site-controlled NW arrays at room temperature, with tunable wavelengths covering the whole telecom band.

To confirm the uniform lasing properties of the NW array, which is a crucial prerequisite to construct the large-scale active nanoscale lasers with stable, reliable and uniform performance for optoelectronic/photonic integrated circuits, PL intensity imaging is further conducted. Figure 4e shows 4 faint luminescence spots from the NW array under low pump fluence. Limited by our optical setup, only 4 NWs can be observed in one image at the same time. Above lasing threshold, these 4 NWs show simultaneous lasing with the emitted coherent light forming speckle fringes, as displayed in Fig. 4f. The corresponding emission spectra, normalized spectral intensity map and L-L curves are also shown in Fig. S16. By placing an attenuator in the collection path to prevent overexposure, 4 bright spots can be observed from the NW top facets shown in Fig. 4g, further validating the good uniformity of NW lasers. To demonstrate the generality of these standing NW lasing, PL images of multiple NWs from different locations within the same array can be found in Fig. S15. In addition, video 1 shows the variations in PL emission from multiple NWs as the pump fluence increased. The video demonstrates the process of PL emission changing from spontaneous emission to stimulated emission as the pump intensity increases, showing that the lasing thresholds of different NWs vary slightly. In conclusion, the collective lasing phenomenon of these NW arrays shows great potential as a promising candidate to produce large scale high density nanolaser sources.

## Discussion

InGaAs/InP core–shell MQW NW arrays have been grown using selective area epitaxy following a carefully designed multi-step growth strategy to control NW length and diameter to achieve uniform morphology, strong carrier confinement, sufficient optical gain, and vertical F–P cavities. Vertical lasing from individual NWs of the vertical arrays is achieved. By adjusting the In composition of the MQWs, the lasing peak can be tuned over a wide range across the telecommunication O-band to C-band window at room temperature. The vertical emission direction, low threshold, high characteristic temperature, as well as uniform lasing simultaneously from a large number of individual NWs within the NW array provide a promising scalable pathway towards cost-effective on-chip advanced optoelectronic and photonic integrated circuits.

## Materials and methods

### Optical experiment method

A confocal photoluminescence microscopy system was used for the optical characterization of the NWs. A frequency-doubled pulsed laser (19.8 MHz, pulse width 11 ps, 532 nm) was used to excite the NWs. The PL emission from the NW was detected by an InGaAs CCD. A linear polarizer was inserted in the signal collection path of the optical system to perform polarization analysis for NW lasing. The carrier lifetime measurement was performed using a time-correlated single photon counting (TCSPC) system composed of an attached single photon detector (SPD, resolution: 50 ps) and a Multi-Channel Scaling (MCS) board (resolution: 25 ps). For low-temperature measurements, a cryostat operating in the range of 4 to 300 K was used.

## Supplementary information

Supplementary information for Telecom-band Multiwavelength Vertical Emitting Quantum Well Nanowire Laser Arrays

Video 1
