# Supplementary material for: Telecom-band multiwavelength vertical emitting quantum well nanowire laser arrays
Source: Light Sci Appl. 2024 Sep 4;13:230. doi: 10.1038/s41377-024-01570-7 (PMC11372134; doi:10.1038/s41377-024-01570-7)
Supplement: Supplementary file 1 — Supplementary information for Telecom-band Multiwavelength Vertical Emitting Quantum Well Nanowire Laser Arrays [file 41377_2024_1570_MOESM1_ESM.pdf]

Supplementary Information for
Telecom-band Multiwavelength Vertical Emitting Quantum Well Nanowire Laser Arrays

Xutao Zhang[1], Fanlu Zhang[2], Ruixuan Yi[3], Naiyin Wang[2], Zhicheng Su[2], Mingwen Zhang[3], Bijun Zhao[3], Ziyuan Li[2], Jiangtao Qu[4], Julie M. Cairney[4], Yuerui Lu[5], Jianlin Zhao[3], Xuetao Gan[3*], Hark Hoe Tan[2, 6], Chennupati Jagadish[2, 6] and Lan Fu[2, 6*]

[1]Frontiers Science Center for Flexible Electronics, Xi'an Institute of Flexible Electronics (IFE) and Xi'an Institute of Biomedical Materials & Engineering, Northwestern Polytechnical University, 127 West Youyi Road, Xi'an 710072, China

[2]Department of Electronic Materials Engineering, Research School of Physics, The Australian National University, Canberra, ACT 2600, Australia

[3]Key Laboratory of Light Field Manipulation and Information Acquisition, Ministry of Industry and Information Technology, and Shaanxi Key Laboratory of Optical Information Technology, School of Physical Science and Technology, Northwestern Polytechnical University, Xi'an 710129, China

[4]Australian Centre for Microscopy and Microanalysis, the University of Sydney, Sydney, NSW 2006, Australia

[5]School of Engineering, College of Engineering, The Australian National University, Canberra ACT 2600, Australia

[6]ARC Centre of Excellence for Transformative Meta-Optical Systems, Research School of Physics, The Australian National University, Canberra, ACT 2600, Australia

1. Growth method 3

2. Comparison of optical properties of InP NWs with different structures 5

3. Summary of growth conditions for different InP-based NWs 6

4. Structural characterization of the MQW NW 9

5. Optical properties of the WZ based InGaAs/InP NW 13

6. Mode simulations 14

7. Electric field distributions of the different transverse modes 15

8. Temperature dependent lasing characterization 16

9. References 19

1. Growth method

Before growth, ~30 nm of SiO_2 was first deposited on InP (111)A substrates at 300 °C by plasma-enhanced chemical vapor deposition. Then hexagonal arrays of holes were patterned by electron beam lithography, followed by reactive ion etching. After trim etching with phosphoric acid and hydrogen peroxide, the substrate was loaded into the MOCVD system (Aixtron CCS 3×2) with a close-coupled shower head reactor for SAE growth. Trimethylindium, trimethylgallium, phosphine and arsine were used as precursors for the In, Ga, P, and As, respectively. To adjust the lasing wavelength, the flux ratio of In/(In+Ga) in the NW quantum wells was increased from 0.45 to 0.6.

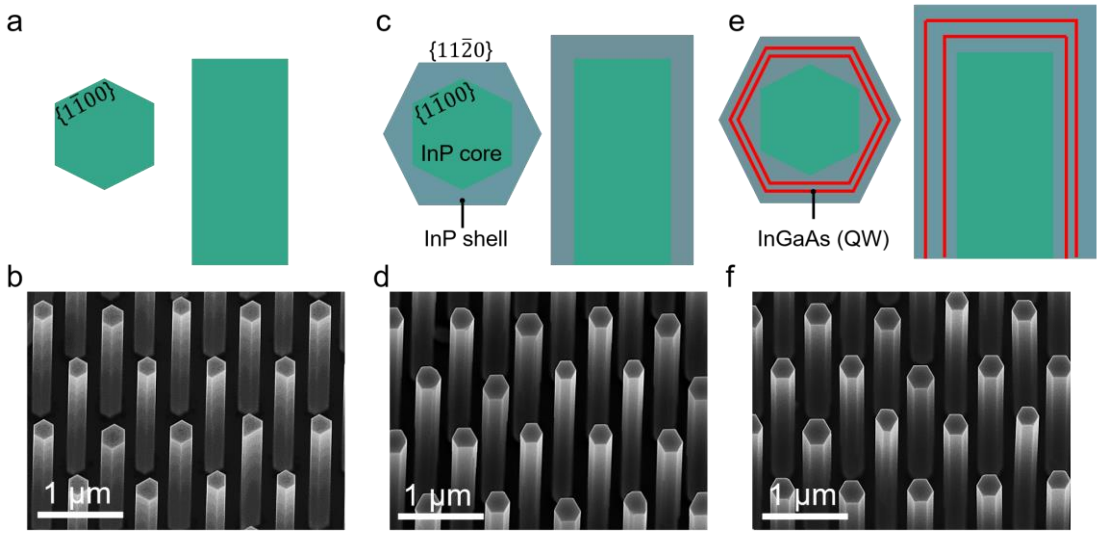

Fig. S1. (a, c, e) Schematics of the lateral (left) and vertical (right) cross-section of WZ based InP nanowire, InP core-shell nanowire, and InGaAs/InP MQW nanowire, respectively, with the corresponding tilted-view SEM images (b, d, f) grown under different conditions. It has been reported that $\{1\bar{1}00\}$ facets of InP nanostructure have lower surface energy at high-temperature condition[1], while $\{11\bar{2}0\}$ facets have lower surface energy at low-temperature condition[1-3]. Since

facets with lower surface energy are more stable, the $\{1\bar{1}00\}$ to $\{11\bar{2}0\}$ facet rotation happens for InP core-shell NW to maintain the lowest possible surface energy during the lower temperature growth step.

2. Comparison of optical properties of InP NWs with different structures

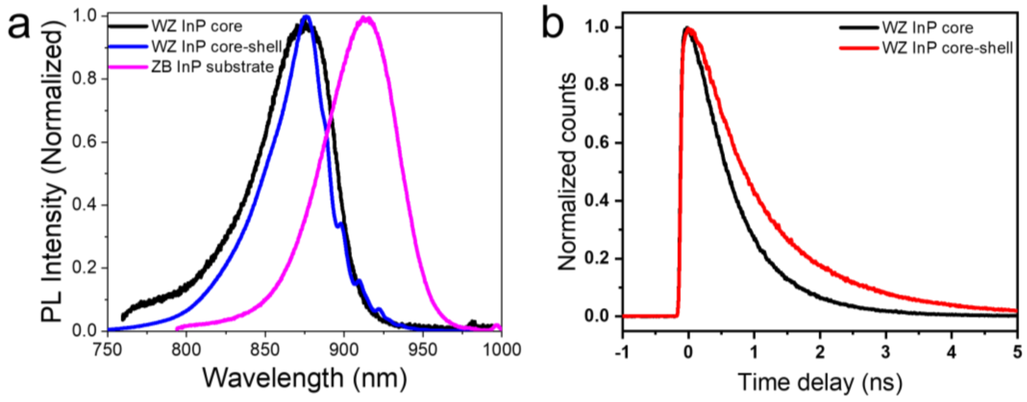

Fig. S2. (a) Normalized PL spectra of WZ InP NW, WZ InP core-shell NW and ZB InP substrate at room temperature. (b) Time-resolved photoluminescence (TRPL) decay curve of WZ InP NW and WZ InP core-shell NW. Both InP core-shell and WZ InP nanowires show the same PL peak located at the wavelength of ~875 nm, whereas the ZB InP substrate exhibits a longer wavelength peak at ~920 nm, indicating that the crystal structure of InP shell is predominantly WZ-based. On the lower energy shoulder of InP core-shell nanowire PL spectra, several smaller and periodic peaks could be observed, which are attributed to the Fabry-Perot cavity effect, as more optical modes can be supported in the InP core-shell nanowire due to their larger diameter. The minority carrier lifetime of the WZ InP nanowire and InP core-shell nanowire can be extracted to be 0.74 and 1.10 ns, by fitting the TRPL decay curve with a single exponential model.

3. Summary of growth conditions for different InP-based NWs

Table S1. Summary of different growth conditions and facet information for WZ, mixed ZB/WZ, and WZ core-shell InP nanowires.

Conditions	i	ii	iii	
	WZ<br>InP nanowire	Mixed ZB/WZ<br>InP nanowire	WZ core-shell<br>InP nanowire	
Growth strategy	one-step growth	one-step growth	two-step growth	
			1st step	2nd step
Facets	$\{1\bar{1}00\}$	$\{1\bar{1}0\}$	$\{1\bar{1}00\}$	$\{11\bar{2}0\}$
Growth Temperature	~ 680°C	~ 615°C	~ 680°C	~ 615°C
TMIn [mol/min]	4.20×10^{-6}	1.26×10^{-6}	4.20×10^{-6}	1.26×10^{-6}
PH3 [mol/min]	1.25×10^{-3}	5.31×10^{-3}	1.25×10^{-3}	5.31×10^{-3}
V/III ratio	297	4214	297	4214
Diameter	hole size dependent	time dependent	time dependent	
Length	0 ~ 15 μm	0 ~ 2 μm	0 ~15 μm	
Stacking fault density	Low	High	Low	
Top-view<br>SEM images	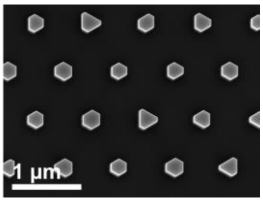	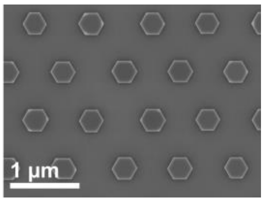	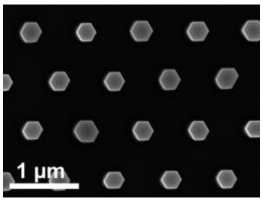	
Tilt-view<br>SEM images	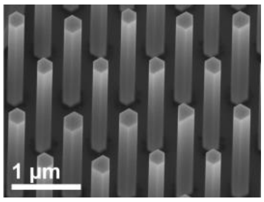	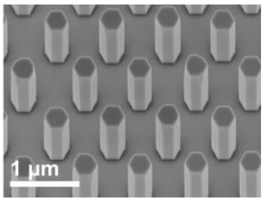	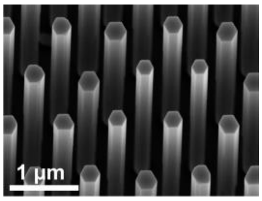	
Schematic	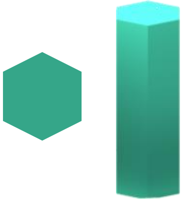	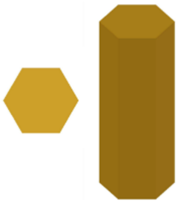	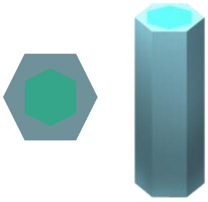	
Reference	4	5	This work	

Table S2. Growth parameters for InGaAs/InP MQW nanowires.

Growth step	Temperature	Time (min)	Repeat (times)	TMIn [mol/min]	TMGa [mol/min]	PH_3 [mol/min]	AsH_3 [mol/min]
InP	~ 680 °C	6	1	4.20×10^{-6}	-	1.25×10^{-3}	-
InP	~ 615 °C	9	1	1.26×10^{-6}		5.31×10^{-3}	
InGaAs QW	~ 615 °C	0.75-1	10	5.08×10^{-7}	2.54×10^{-6}	-	5.31×10^{-3}
InP Barrier	~ 615 °C	2-3	10	1.26×10^{-6}	-	5.31×10^{-3}	-
InP	~ 615 °C	1	1	1.26×10^{-6}	-	5.31×10^{-3}	-

Table S3. Summary of the various properties of different InGaAs/InP MQW nanowire structures achieved under different growth strategies.

Materials	InGaAs/InP MQW	InGaAs/InP single-QW	InGaAs/InP MQW	InGaAs/InP MQW
SEM images	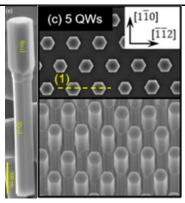	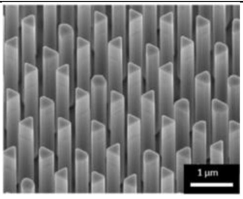	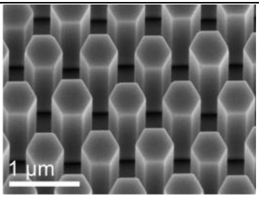	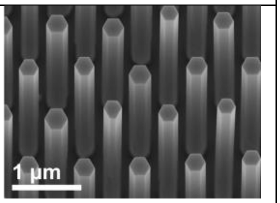
Facet transition	$\{1\bar{1}00\}$ to multiple facets	$\{1\bar{1}00\}$	$\{110\}$	$\{1\bar{1}00\}$ to $\{11\bar{2}0\}$
Morphology transition	Hexagonal to multi-facet	Hexagonal to triangular	Hexagonal	Hexagonal to hexagonal
Crystal structure	WZ	WZ	Mixed ZB/WZ	WZ
Stacking fault density	Low	Low	High	Low
Length	0 – 15 µm (Time controlled)	0 – 15 µm (Time controlled)	0 – 2 µm (maximum ~2 µm)	0 – 15 µm (Time controlled)
Diameter	-	-	Time controlled	Time controlled
Notes	Non-uniform	Wrinkled surface	Highly uniform	Highly uniform
Reference	[6]Reproduced with permission. Copyright 2018, American Chemical Society	[7]Reproduced with permission. Copyright 2020, Elsevier Ltd.	[5]Reproduced with permission. Copyright 2022, John Wiley & Sons, Ltd.	This work

4. Structural characterization of the MQW NW

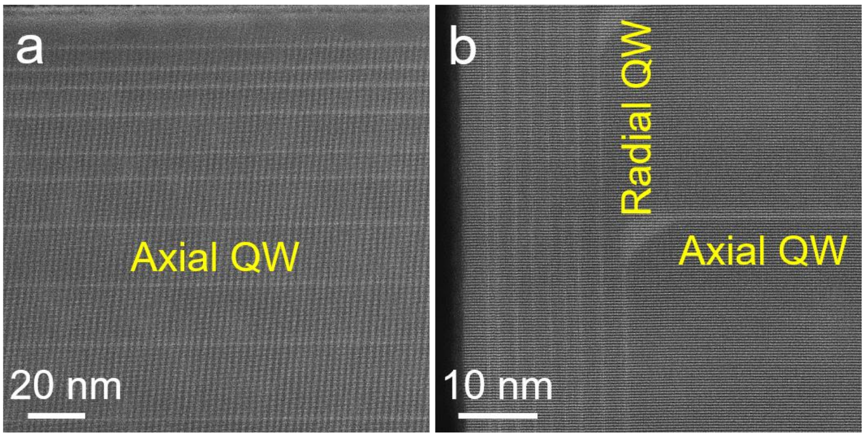

Fig. S3 STEM-HAADF image taken along [112] zone axis from the vertical cross-section of nanowire top segment, showing the growth of axial QWs (a) and the radial QWs (b).

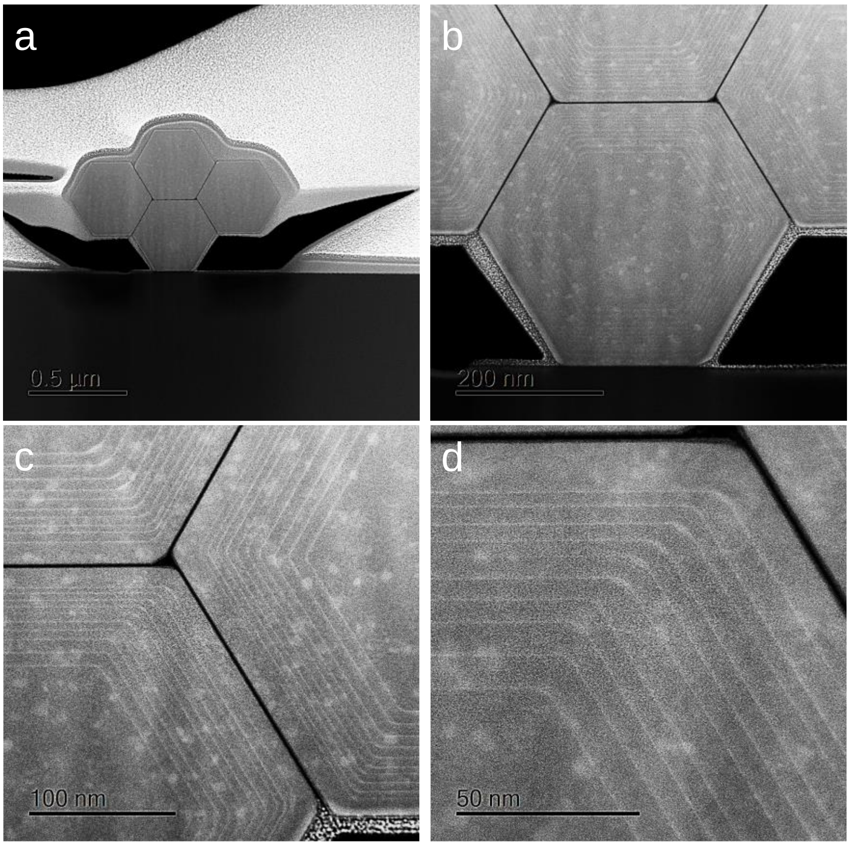

Fig. S4 STEM-HAADF images of the lateral cross-section of four different InGaAs/InP QW NWs at different magnifications, showing a uniform hexagonal prism shape.

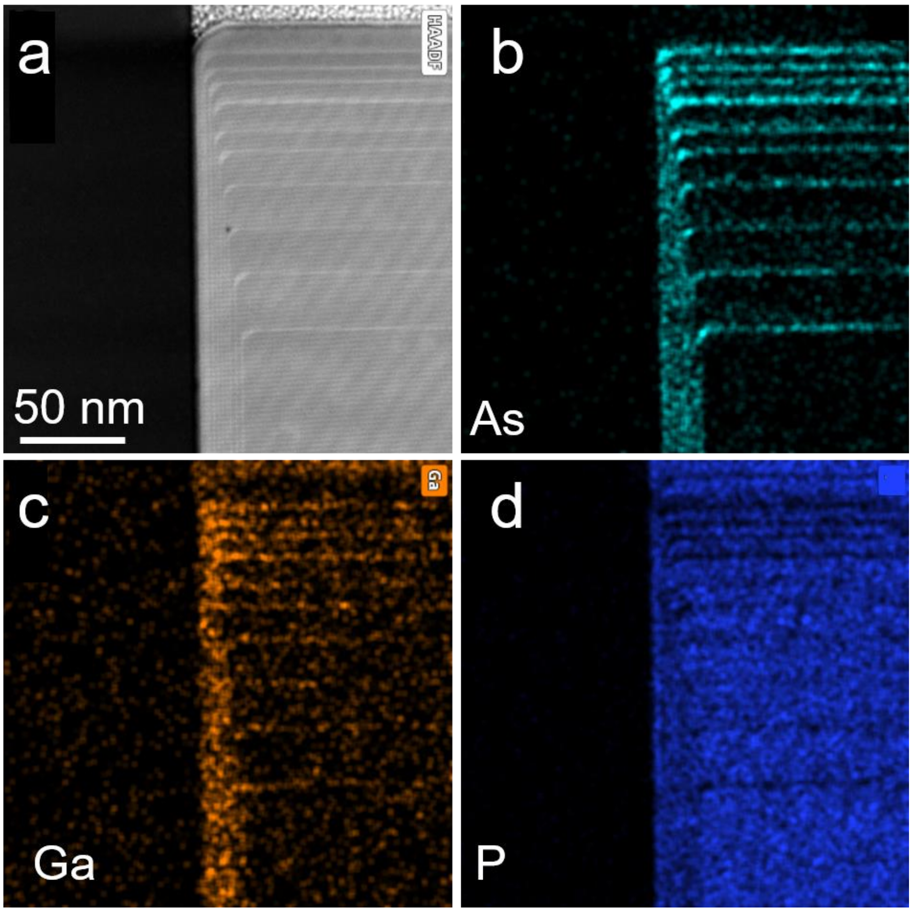

Fig. S5 Structural characterization of InP/InGaAs MQW NW 1. HAADF image of the NW cross-section and corresponding EDS As, Ga and P maps.

Here, we note a high concentration of P atoms in the QWs, indicating quaternary InGaAsP formation instead of intended InGaAs growth. Some residual Ga and As atoms can also be observed in the InP barrier layers, suggesting atomic interdiffusion (In-Ga and As-P) at the QW/barrier interface. On the other hand, the Ga concentration is much lower than In concentration in the QW layers, even though a much higher molar fraction of Ga precursor (that of than In precursor) was used during the growth, which may be attributed to the shorter diffusion length of Ga atoms compared with that of the In atoms.

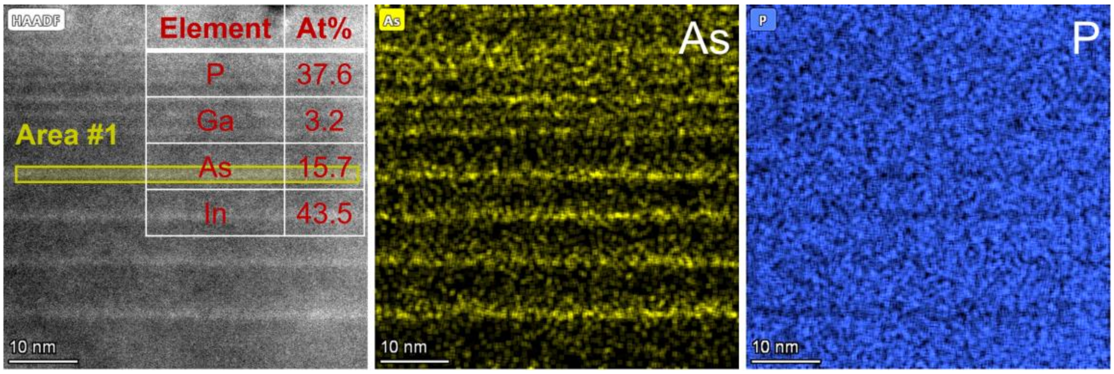

Fig. S6 EDX maps of the NW lasing at 1550 nm. The QW region shown in the Area #1 of HAADF image has a P/Ga/As/In ratio of 37.6:3.2:15.7:43.5.

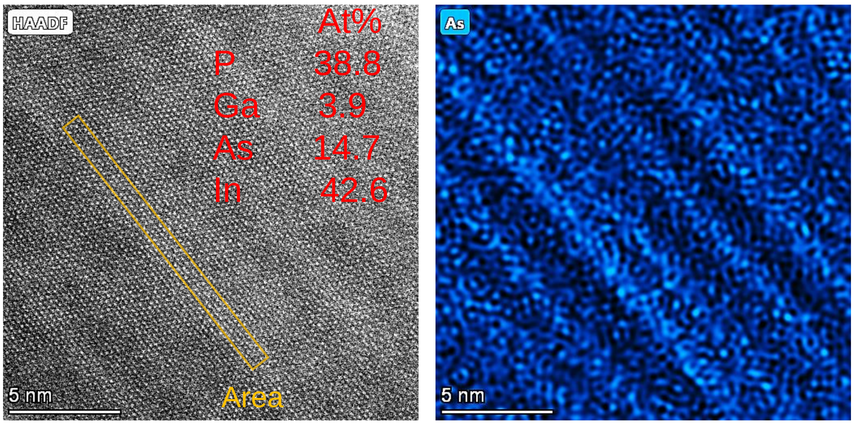

Fig. S7 EDX maps of the NW lasing at 1365 nm. The QW region shown in the Area #1 of HAADF image has a P/Ga/As/In ratio of 38.8:3.9:14.7:42.6.

5. Optical properties of the WZ based InGaAs/InP NW

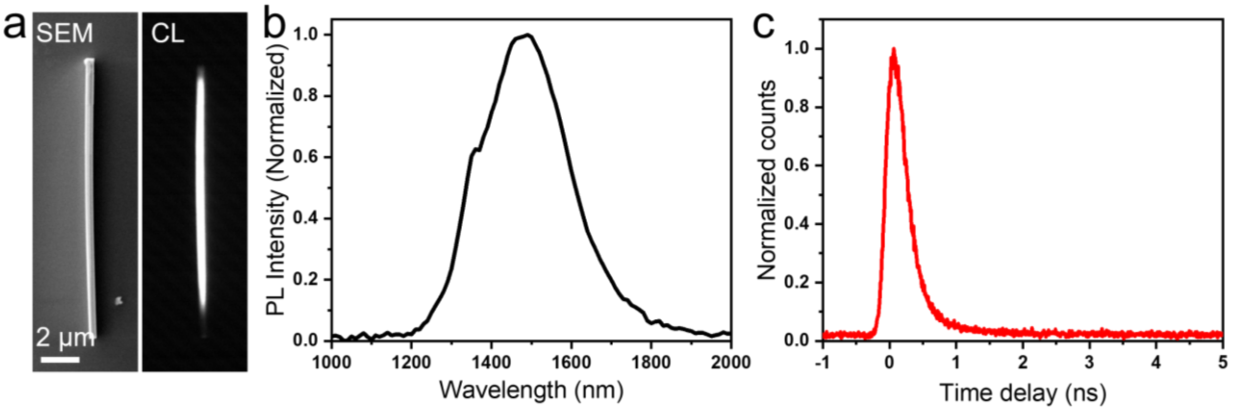

Fig. S8 (a) SEM and pan-chromatic (1.0-1.6 μm) CL image of WZ based InGaAs/InP 10-QW nanowire. (b) PL spectrum and TRPL decay curve (c) directly measured from the top of the nanowire array.

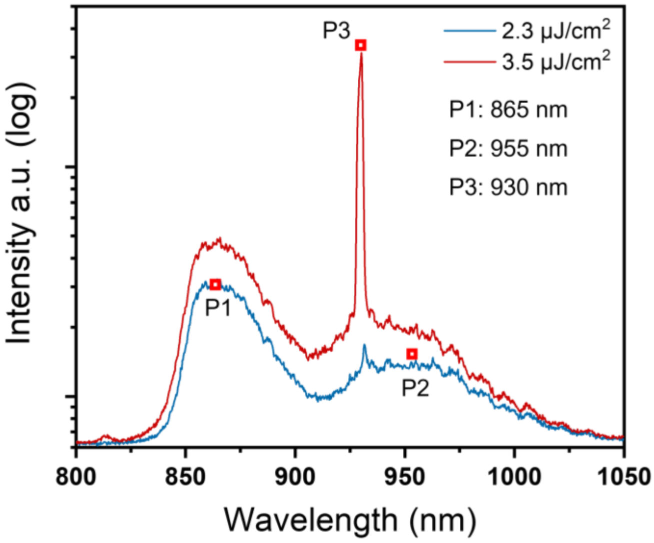

Fig. S9 PL emission spectra under different pump fluences. Among them, P1 (emission from InP) and P2 (emission from the QWs) are in the amplified spontaneous emission regime (0.85 P_{th}) and P3 is in the stimulated emission (1.3 P_{th}).

6. Mode simulations

The two-dimensional (2D) finite difference eigenmode (FDE) solver was used to calculate the transverse modes supported in the as-grown NW. The model with an InP NW standing on the SiO_2 substrate with a 120 nm hole opening was used. A square shaped 2D FDE solver region was set at the center of the cross-section of NW (Size: 5 times of NW diameter; Boundary setting: Metal).

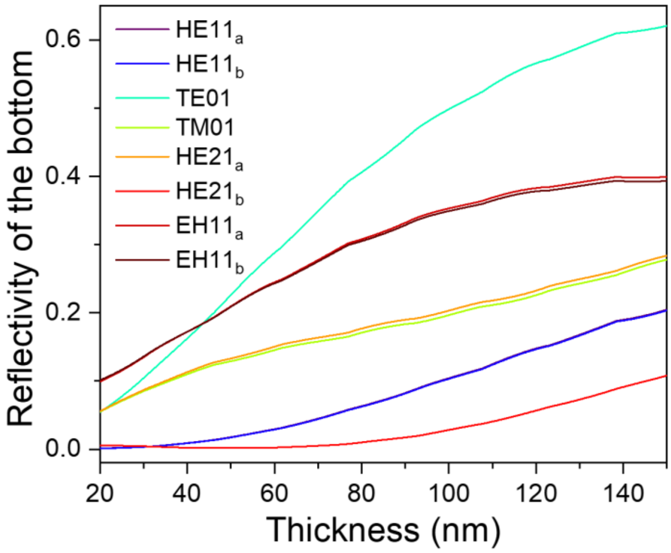

Fig. S10 Bottom surface reflectivity versus the thickness of SiO_2 for each transverse mode of the NW with a diameter of 405 nm, showing that the bottom surface reflectivity for each transverse mode increase with the thickness of SiO_2. Even for the NW with 30 nm SiO_2 layer deposited on top of the InP substrate, reasonable reflectivity can still be obtained for the TE_{01}, HE_{21a}, HE_{21b}, and EH_{11a} and EH_{11b} modes.

7. Electric field distributions of the different transverse modes

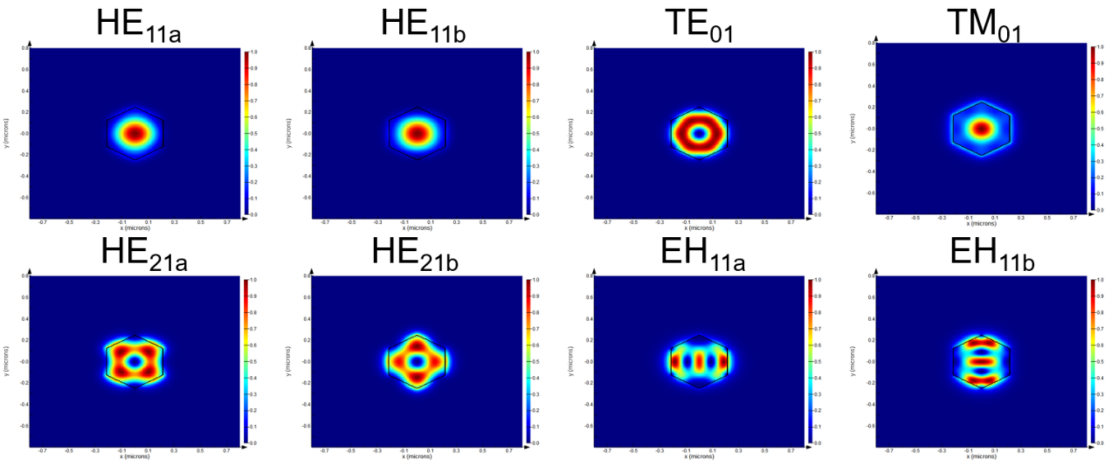

Fig. S11 Simulated electric field distributions of the different transverse modes.

8. Temperature dependent lasing characterization

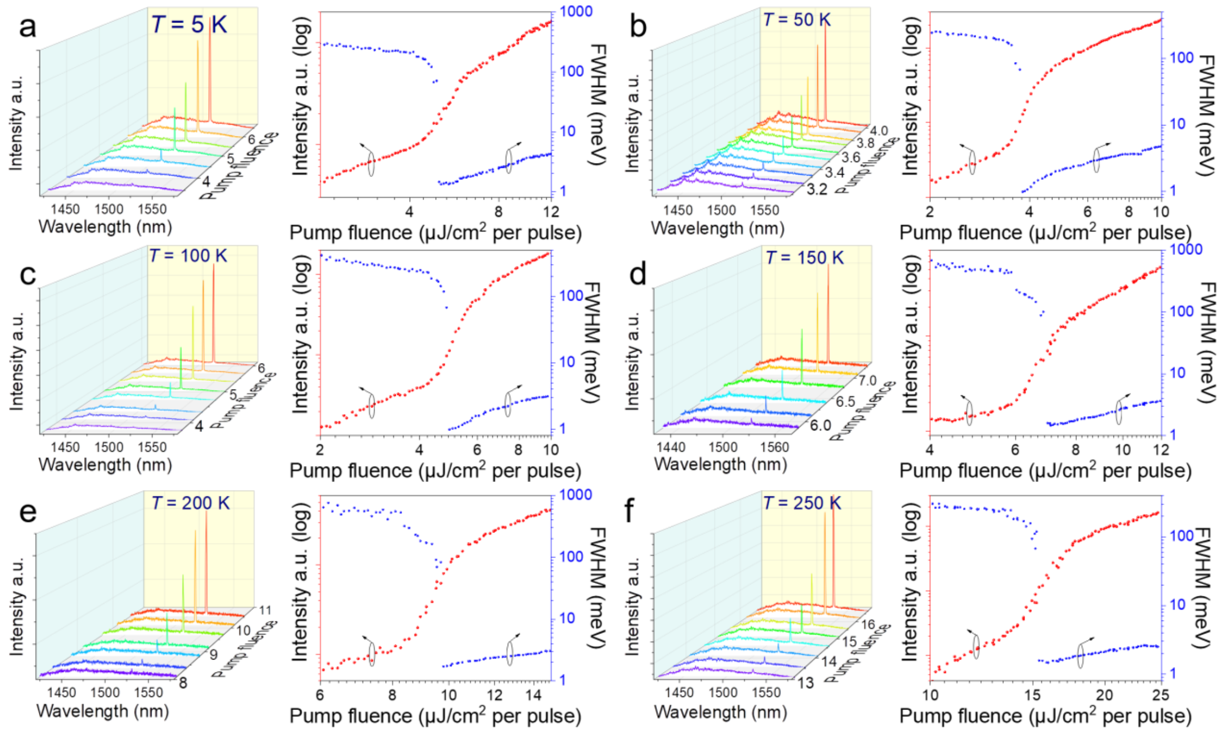

Fig. S12 Lasing characterization of a standing NW at different temperatures. Emission spectra and corresponding lasing emission intensity (red dots) and the full width at half maximum (FWHM) of the spectrum (blue dots) as a function of pump fluence at 5 K (a), 50 K (b), 100 K (c), 150 K (d), 200 K (e), 250 K (f).

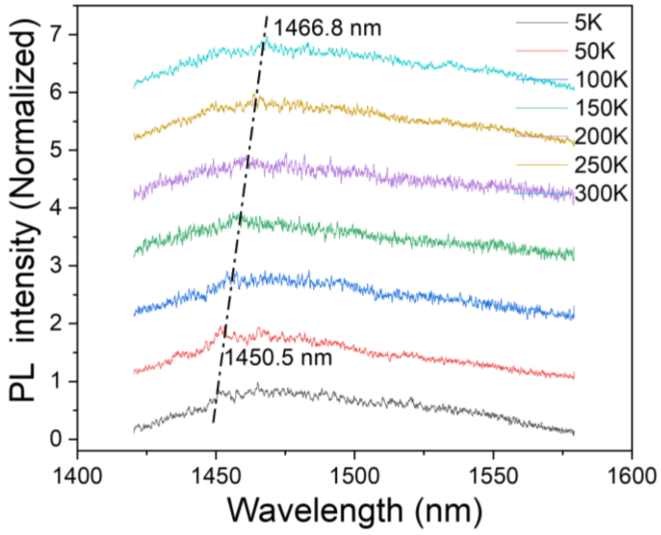

Fig. S13 Temperature dependent PL intensities under weak pump fluence, showing a slight red shift of ~ 15 nm in band gap when the temperature was raised from 5 to 300 K.

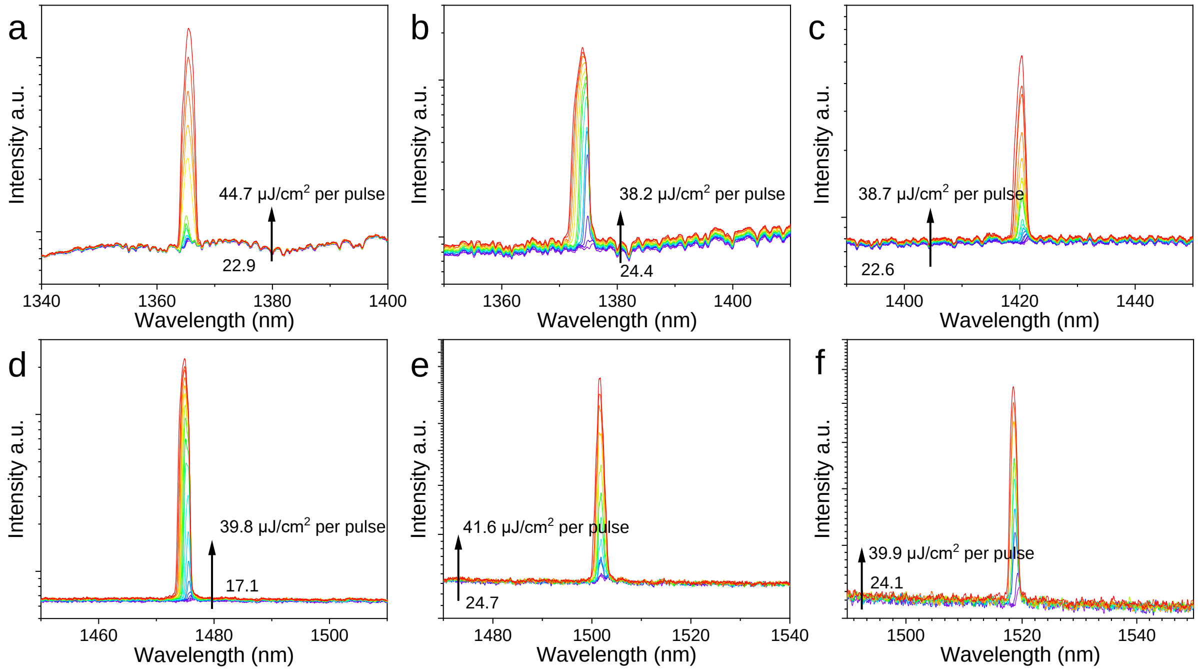

Fig. S14 Telecom-band emission spectra at different pump fluences from different NWs.

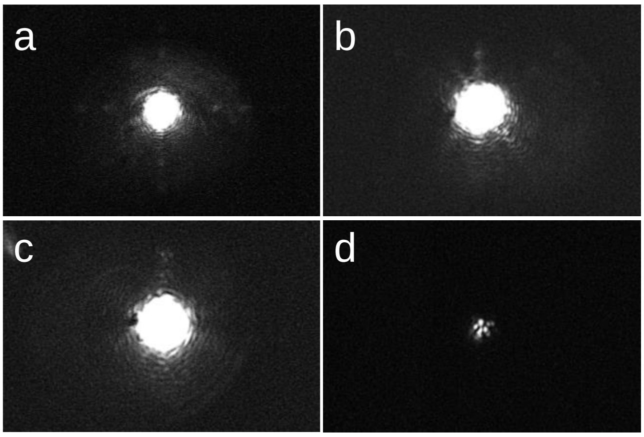

Fig. S15 PL images from three other locations in the NW array after lasing threshold (a-c). (d) An attenuated image on (c).

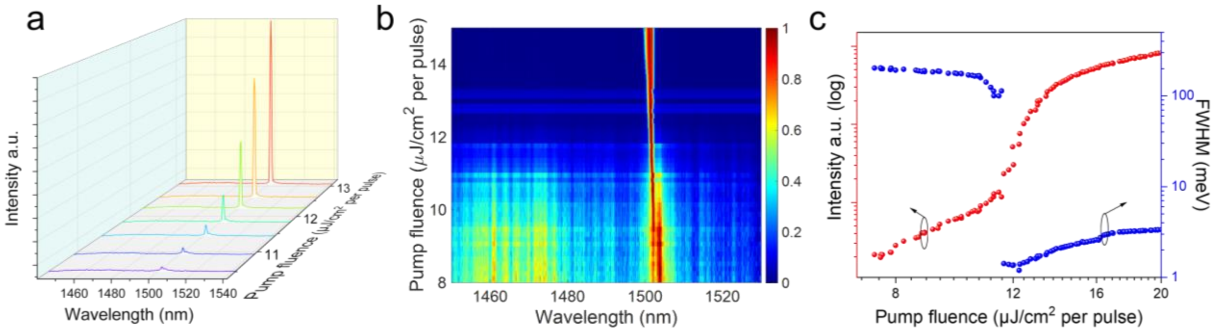

Fig. S16. Lasing characterization form 4 NWs at 10 K. (a) Emission spectra at different pump fluences and (b) the corresponding normalized spectral intensity map. (c) Emission intensity (red) and the corresponding FWHM (blue) of the spectra versus pump fluence.

9. References

1 Wang, N. *et al.* Shape engineering of InP nanostructures by selective area epitaxy. *ACS Nano* **13**, 7261-7269, (2019).

2 Fahed, M., Desplanque, L., Coinon, C., Troadec, D. & Wallart, X. Impact of P/In flux ratio and epilayer thickness on faceting for nanoscale selective area growth of InP by molecular beam epitaxy. *Nanotechnology* **26**, 295301, (2015).

3 Zhang, X. *et al.* Growth mechanism of InP nanostructure arrays by self-catalyzed selective area epitaxy: a deep understanding of thermodynamic and kinetic theories. *Cryst. Growth Des.* **21**, 988-994, (2021).

4 Gao, Q. *et al.* Selective-area epitaxy of pure wurtzite InP nanowires: high quantum efficiency and room-temperature lasing. *Nano Lett.* **14**, 5206-5211, (2014).

5 Zhang, F. L. *et al.* A new strategy for selective area growth of highly uniform InGaAs/InP multiple quantum well nanowire arrays for optoelectronic device applications. *Adv. Funct. Mater.* **32**, 2103057, (2022).

6 Yang, I. *et al.* Radial growth evolution of InGaAs/InP multi-quantum-well nanowires grown by selective-area metal organic vapor-phase epitaxy. *ACS Nano* **12**, 10374-10382, (2018).

7 Yang, I. *et al.* Highly uniform InGaAs/InP quantum well nanowire array-based light emitting diodes. *Nano Energy* **71**, 104576, (2020).
